# Multilocus sequence typing of *Enterocytozoon bieneusi* in crab-eating macaques (*Macaca fascicularis*) in Hainan, China

**DOI:** 10.1186/s13071-020-04046-w

**Published:** 2020-04-08

**Authors:** Li Chen, Na Li, Yaqiong Guo, Jianguo Zhao, Yaoyu Feng, Lihua Xiao

**Affiliations:** 1 State Key Laboratory of Bioreactor Engineering, School of Resource and Environmental Engineering, East China University of Science and Technology, Shanghai, 200237 China; 2 Key Laboratory of Zoonosis of Ministry of Agriculture, College of Veterinary Medicine, South China Agricultural University, Guangzhou, 510642 China; 3 Guangdong Laboratory for Lingnan Modern Agriculture, Guangzhou, 510642 China; 4 Key Laboratory of Tropical Biological Resources of Ministry of Education, School of Life and Pharmaceutical Sciences, Hainan University, Haikou, 570228 Hainan China

**Keywords:** *Enterocytozoon bieneusi*, Multilocus sequence typing, Crab-eating macaques, Population structure, Subpopulation

## Abstract

**Background:**

*Enterocytozoon bieneusi* is one of common intestinal pathogens in humans and animals including non-human primates (NHPs). Many zoonotic pathogens including *E. bieneusi* have been found in these animals. However, there are few studies on the population structure of *E. bieneusi* in NHPs. To infer the gene diversity and population genetics of *E. bieneusi*, we selected 88 *E. bieneusi-*positive samples from crab-eating macaques for multilocus characterizations in this study.

**Methods:**

The *E. bieneusi* isolates examined belonged to three common genotypes with different host ranges by sequence analysis of the ribosomal internal transcribed spacer (ITS): Type IV (*n* = 44), Macaque3 (*n* = 24) and Peru8 (*n* = 20). They were further characterized by sequence analysis at four microsatellite and minisatellite loci (MS1, MS3, MS4 and MS7). DnaSP, Arlequin and LIAN were used to analyze the sequence data together with those from the ITS locus to infer the population genetics. Subpopulation structure was inferred using phylogenetic and STRUCTURE analyses.

**Results:**

Seventy-two (81.8%), 71 (80.7%), 76 (86.4%) and 79 (89.8%) samples were amplified and sequenced successfully at the MS1, MS3, MS4 and MS7 loci, respectively, with 53 having sequence data at all five MLST loci including ITS. Altogether, 33 multilocus genotypes (MLGs) were produced based on concatenated sequences from the 53 samples. In phylogenetic analyses of sequences and allelic data, four major subpopulations (SPs) were observed with different ITS genotypes in each of them: Type IV and Peru8 in SP1 and SP2; Type IV, Macaque3 and Peru8 in SP3; and Type IV and Macaque3 in SP4. SP3 and SP4 were phylogenetically related and might be NHP-specific based on the fact that Macaque3 is mostly found in NHPs. A strong linkage disequilibrium (LD) was observed among the multilocus sequences and allelic data.

**Conclusions:**

The significant LD in the multilocus sequence analysis indicated the presence of an overall clonal population structure of *E. bieneusi* in crab-eating macaques. The inconsistent segregation of MLGs among ITS genotypes suggested some occurrence of genetic recombination. These observations should improve our understanding of the population genetics of *E. bieneusi* in NHPs.
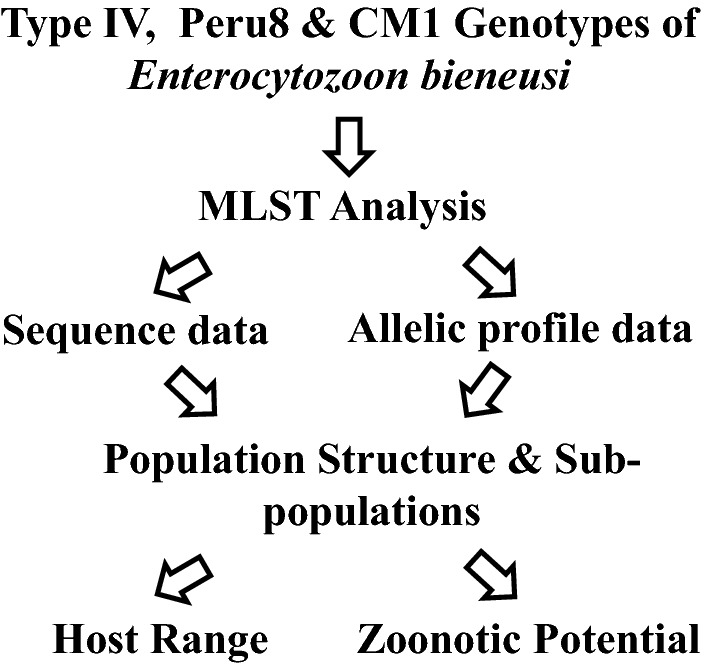

## Background

*Enterocytozoon bieneusi* is an obligate intracellular pathogen infecting humans, domestic animals and wildlife [1]. Since the detection of *E. bieneusi* in AIDS patients in 1985 for the first time, this pathogen has been found in other immunocompromised/immunodeficient individuals, such as organ transplant recipients, children, and life-threatening and persistent diarrhea often occur in these populations [2–7]. It has been recently identified in several species of non-human primates (NHPs) in China and elsewhere [8–14].

Approximately 500 *E. bieneusi* genotypes have been identified based on sequence analysis of the internal transcribed spacer (ITS) region of the rRNA gene [15, 16]. They belong to 11 phylogenetic groups. Among them, ITS genotypes in Group 1 and Group 2 have been found in a broad range of hosts including humans and are probably responsible for most zoonotic or cross-species *E. bieneusi* infections whereas host adaptation seems to be more common in genotypes of Groups 3 to 11 according to a recent review of *E. bieneusi* [6]. To date, more than 100 *E. bieneusi* genotypes have been detected in NHPs, mostly belonging to Group 1 [6, 9, 10, 13, 14, 17–21]. Among them, Type IV and Peru8 are common ITS genotypes in humans and various animals in many countries, including China [22]. Conversely, Macaque3 has been mostly found in monkeys and occasionally in dogs in China [1, 21, 23–26].

Multilocus sequence typing (MLST) based on sequence analysis of four markers (MS1, MS3, MS4 and MS7) with simple tandem repeats and the ITS has been used in investigating the transmission dynamics of *E. bieneusi* in humans and some animals, including pigs, sheep, rabbits, calves, black bears, giant pandas and horses [27–35]. Thus far, only three studies have used the MLST analysis to characterize *E. bieneusi* isolates in NHPs, and only one study has found the presence of an overall clonal population structure and an epidemic population structure in subpopulations of this pathogen in NHPs [11, 36, 37].

In this study, 88 *E. bieneusi*-positive samples from crab-eating macaques (*Macaca fascicularis*) in Hainan Province, China were used for MLST analysis and population genetic characterization of *E. bieneusi*. The present study aims at evaluating the genetic diversity and population structure of *E. bieneusi* in NHPs to better understand the transmission and epidemiology of *E. bieneusi* in these animals.

## Methods

### Source of *E. bieneusi*-positive samples

The 88 *E. bieneusi-*positive samples used in this study were from crab-eating macaques in Hainan, China. They consisted of ITS genotypes Type IV (*n* = 44), Macaque3 (*n* = 24) and Peru8 (*n* = 20), which were identified by sequence analysis of the ITS region of the rRNA gene in a previous study [8]. They were collected from different animals and used for further molecular characterization in this study. Other minor *E. bieneusi* genotypes from that study were not included in the present study, as the small numbers of samples for each of them made examinations of intra-genotypic variations of them difficult.

### MLST analysis of *E. bieneusi*

Four established MLST loci were targeted in this study, including three microsatellite loci (MS1, MS3 and MS7) and one minisatellite locus (MS4). These loci were amplified by nested PCR using published primers and conditions [32]. A positive control (DNA of genotype BEB4 which usually infects cattle rather than NHPs) and a negative control (reagent water without DNA) were used in all PCR tests. PCR was performed twice on each DNA sample at each genetic locus. The secondary PCR products generated were bi-directionally sequenced on an ABI 3730 Genetic Analyzer (Applied Biosystems, Foster City, CA). The nucleotide sequences obtained were assembled using software ChromasPro 1.32 (http://technelysium.com.au/ChromasPro.html), and aligned with each other and homologous sequences downloaded from GenBank using ClustalX (http://clustal.org). The sequence alignments at the four genetic loci (MS1, MS3, MS4 and MS7) were used in the assessment of sequence polymorphism within the ITS genotypes, determination of MLGs and phylogenetic analyses of the MLST data.

### Population genetic analysis of sequence data

The multilocus sequences data (concatenated sequences from all five markers, including the ITS) generated were used in the analysis of the population genetics of *E. bieneusi* using the software DnaSP version 5.10.01 (http://www.ub.edu/dnasp/) [38]. The population structure of *E. bieneusi* was assessed by intragenic and intergenic linkage disequilibrium (LD), neutrality (Fu’s statistics or Fs values on selective neutrality based on genotype frequency), and recombination events (Rms).

### Population genetic analysis of allelic profile data

Pairwise intergenic LD based on allelic profiles at the five genetic loci was evaluated using the exact test and Markov chain parameters implemented in Arlequin version 3.5.1.2 [39]. The standardized index of association (*I*^S^_A_) was measured using LIAN 3.7 [40].

### Subpopulation structure analysis

The phylogenetic relationship among MLGs including both SNPs and INDELs was assessed using the maximum likelihood (ML) analysis and general time reversible model implemented in MEGA 6 (https://www.megasoftware.net). The reliability of clusters formed was assessed using bootstrap analysis with 1000 replicates. An un-weighted pair group mean average (UPGMA) tree of the allelic data was generated using MEGA 6. Wright’s fixation index (*F*_ST_) of *E. bieneusi* isolates was calculated using software Arlequin version 3.5.2.2.

STRUCTURE version 2.3.1 was used to identify distinct sub-populations, with *K*-values of 2–4 [41]. *F*_ST_ was calculated to assess the robustness of the sub-structuring and to determine genetic distances between subpopulations.

## Results

### PCR amplification efficiency at the MLST loci

The PCR amplification efficiencies were 81.8%, 80.7%, 86.4% and 89.8% at the MS1, MS3, MS4 and MS7 loci, respectively (Table 1). For ITS genotypes Type IV and Peru8, the amplification efficiencies were similar at the MS1 (88.7% and 80.0%), MS3 (81.8% and 85.0%), MS4 (86.4% and 85.0%) and MS7 (91.2% and 95.0%) loci. For Macaque3, however, the amplification efficiencies at the MS1 (70.8%), MS3 (75.0%), MS4 (87.5%) and MS7 (79.2%) loci were correspondingly lower than those of ITS genotypes Type IV and Peru8 at the three loci except MS4.

**Table 1:** PCR amplification efficiency of DNA from four *Enterocytozoon bieneusi* ITS genotypes at the other four MLST loci

ITS genotype	*n*	No. of specimens amplified/No. of specimens analyzed (%)			
		MS1	MS3	MS4	MS7
Type IV	44	39/44 (88.7)	36/44 (81.8)	38/44 (86.4)	41/44 (91.2)
Macaque3	24	17/24 (70.8)	18/24 (75.0)	21/24 (87.5)	19/24 (79.2)
Peru8	20	16/20 (80.0)	17/20 (85.0)	17/20 (85.0)	19/20 (95.0)
Total	88	72/88 (81.8)	71/88 (80.7)	76/88 (86.4)	79/88 (89.8)

*Abbreviation*: n, number of samples

### Multilocus genotypes

Genetic diversity at the MLST loci was in the form of both SNPs and indels, with 24, 5, 25, 4 and 3 segregation sites at the MS1, MS3, MS4, MS7 and ITS loci, respectively (Table 2). Altogether, 6, 4, 6, 2 genotypes were detected at the MS1, MS3, MS4 and MS7 loci, respectively. The intragenic LD among segregating sites for each locus was calculated using the linear regression equation. The MS3, MS7 and ITS loci were in complete linkage disequilibrium (LD = 1). In contrast, the remaining two loci (MS1 and MS4) had incomplete LD, indicating the potential presence of genetic recombination. This was confirmed by further assessment of intragenic recombination, which showed the presence of two and five Rms at the MS1 and MS4 loci, respectively.

**Table 2:** Intragenic linkage disequilibrium and recombination events in individual genetic loci among 53 *E. bieneusi* samples

Marker	No. of types	S	P	F	B	LD (|*D′*|)	Rm
MS1	6	24	253	3	0	Y = 0.9838 + 0.0293X	2
MS3	4	5	10	0	0	1	0
MS4	6	25	276	0	0	Y = 0.7859 + 0.0126X	5
MS7	2	4	6	0	0	1	0
ITS	3	3	3	0	0	1	0

*Abbreviations*: S, number of segregating sites; P, number of pairwise comparisons; F, number of significant pairwise comparisons by Fisher’s exact test; B, number of significant comparisons after Bonferroni correction; LD (|*D′*|), linkage disequilibrium per site; Rm, minimum number of recombination events

Of the 88 samples analyzed by MLST, 53 were positive at all four genetic loci. To determine the MLGs, sequences of the five genetic loci (MS1, MS3, MS4, MS7 and ITS) from each of the 53 samples were concatenated. Genetic diversity was assessed based on both total polymorphic sites (finite population variance estimates) and segregating sites only (infinite population variance estimates). Together they formed 33 MLGs with an Hd of 0.98 (Table 3). The mean numbers of pairwise differences in polymorphic and segregating sites were 16 and 14, respectively. The frequency of the MLGs ranged from 9.4% (one MLG with five samples), 7.5% (four MLGs each with three samples), 3.8% (eight MLGs each with two samples), to 1.9% (20 MLGs each with one sample) (Table 4).

**Table 3:** Genetic diversity in 53 *Enterocytozoon bieneusi* samples based on the analysis of concatenated multilocus sequences (2661 bp in length)

Test model	Variability of multilocus sequences										
	No. MLGs	Hd	*k*	Pi	Theta (k)	Fs (obs)	*P* (Fs ≤ obs)	Z_n_*s* (obs)	*P* (Z_n_*s* ≤ obs)	LD (|*D′*|)	Rm
Polymorphic sites	33	0.98	16	0.009	16	− 5.56	0.158				
Segregating sites	33	0.98	14	0.008	14	− 6.91	0.122	0.185	0.976	Y = 0.8679 − 0.2781X	10

*Abbreviations*: MLG, multilocus genotype; Hd, gene diversity; k, mean number of pairwise differences; Pi, nucleotide diversity (average over loci); theta (k), genetic variance based on the mean number of pairwise differences; Fs, Fu’s statistic testing selective neutrality based on genotype frequency; Obs, observed value; P (Fs ≤ Obs), probability of obtaining Fs values equal or lower than the observed; Z_n_*s* (obs), observed Z_n_*s* value; P (Z_n_*s* ≤ Obs), probability of obtaining Z_n_*s* values equal or lower than the observed; LD (|*D*′|), linkage disequilibrium per site; Rm, minimum number of recombination events

**Table 4:** Multilocus sequence types of ITS genotypes Type IV and Macaque3 and Peru8 of *Enterocytozoon bieneusi* in crab-eating macaques

MLG	ITS genotype	Sequence type^a^				No. of individuals
		MS1	MS3	MS4	MS7	
T1	Type IV	3	3	2	1	3
T2		1	1	2	1	1
T3		6	2	1	1	2
T4		1	2	3	1	2
T5		3	2	1	1	1
T6		2	2	2	1	1
T7		4	1	2	1	2
T8		3	1	1	1	2
T9		3	2	2	1	3
T10		2	3	1	1	1
T11		3	1	2	1	5
T12		4	2	2	1	1
T13		5	3	4	2	1
T14		2	3	2	1	1
T15		5	1	1	1	1
T16		2	3	4	2	1
T17		3	1	3	2	1
T18		5	1	2	1	1
C1	Macaque3	2	3	2	2	2
C2		2	1	2	2	2
C3		3	2	2	1	3
C4		1	1	2	1	2
C5		3	4	6	1	1
C6		3	1	2	1	1
C7		3	4	2	1	1
P1	Peru8	3	2	2	1	3
P2		1	2	2	1	1
P3		3	1	2	1	1
P4		1	1	2	1	1
P5		3	2	1	1	2
P6		2	2	1	1	1
P7		2	4	5	2	1
P8		3	4	5	1	1
Total		–	–	–	–	53

^a^All four MLST loci amplified successfully were taken into consideration

### Population structure

Inter-locus LD over all segregating sites within the multilocus contig was assessed for all 53 samples. This produced a Z_n_*s* value of 0.185; the probability *P-*values for expected *Z*_n_*s* ≤ 0.185 was 0.976. The incomplete LD among these 53 samples (|*D′*| Y = 0.8679 − 0.2781X) indicated a possible occurrence of genetic recombination, which was confirmed by results of the recombination analysis (10 Rms) (Table 3). Thereafter, we used Fu’s statistic test to evaluate the genotype frequency in this population. In this test, we obtained Fs values of -5.56 (*P* (Fs < − 5.56) = 0.158) and − 6.91 (*P* (Fs < − 6.91) = 0.122) based on polymorphic sites and segregating sites, respectively (Table 3). The results of Fu’s statistic tests indicated the presence of an excessive number of alleles.

We calculated *I*^S^_A_ to assess the LD between alleles in pairwise combinations of genetic loci [39]. Results of this analysis of allelic profile data showed a positive *I*^S^_A_ value together with *V*_*D*_ > *L*. Furthermore, a significant *P*_*MC*_ value (< 0.001) was obtained using the Monte Carlo method based on the null hypothesis of panmixia (Table 5). All these results indicated that the population was in LD. However, values of *V*_*D*_ < *L* and *P*_*MC*_ > 0.001 were obtained for subpopulations identified in this study or ITS genotype. Thus, these subpopulations and ITS genotypes were in linkage equilibrium (LE) and had the epidemic population structure.

**Table 5:** Results of linkage disequilibrium analysis of allelic profile data

Population^a^	*n*	*H*	*I*^*S*^_*A*_	*P*_*MC*_	*V*_*D*_	*L*	*V*_*D*_ > *L*
All	33	0.6042 ± 0.1024	0.0781	< 0.001	1.0253	0.9422	Y
SP 1	5	0.5600 ± 0.1600	0.0432	0.487	0.8444	1.1362	N
SP 2	9	0.4667 ± 0.1325	− 0.0741	0.908	0.6286	1.3191	N
SP 3	15	0.4549 ± 0.1400	0.0126	0.604	0.8904	1.0392	N
SP 4	4	0.3200 ± 0.1497	− 0.0590	1.000	0.4889	1.0765	N
Type IV	18	0.5020 ± 0.1542	0.0394	0.319	0.8962	0.9065	N
Macaque3	7	0.4476 ± 0.1425	0.0484	0.365	0.9905	1.2423	N
Peru8	8	0.4786 ± 0.1497	0.0820	0.044	1.0622	1.1025	N

^a^Considering each group of isolates with the same multilocus genotype (MLG) as one individual

*Abbreviations*: *n*, number of isolates; H, mean genetic diversity; *I*^*S*^_*A*_, standardized index of association calculated using the program LIAN 3.7; *P*_*MC*_, significance for obtaining this value in 1000 simulations using the Monte Carlo method; *V*_*D*_, variance of pairwise differences; *L*, 95% critical value for *V*_*D*_; *V*_*D*_ > *L* indicates linkage disequilibrium; Y, yes; N, no

### Subpopulation structure

**Fig. 1:**
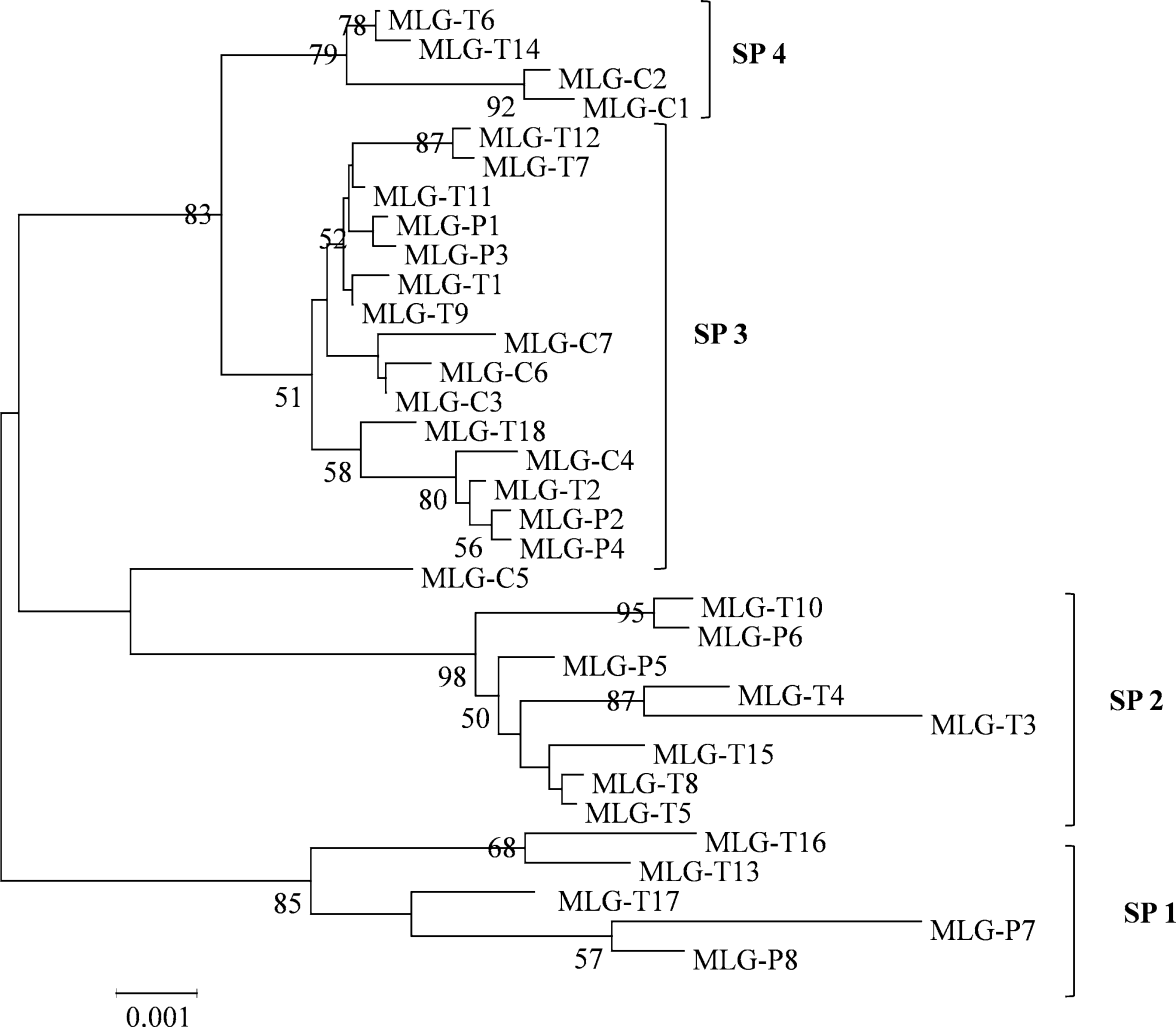
Phylogenetic relationships of multilocus genotypes (MLGs) of ITS genotypes Type IV, Macaque3 and Peru8 of *Enterocytozoon bieneusi* inferred using the maximum likelihood analysis of concatenated ITS, MS1, MS3, MS4 and MS7. Bootstrap values greater than 50% from 1000 replicates are shown at the nodes

**Fig. 2:**
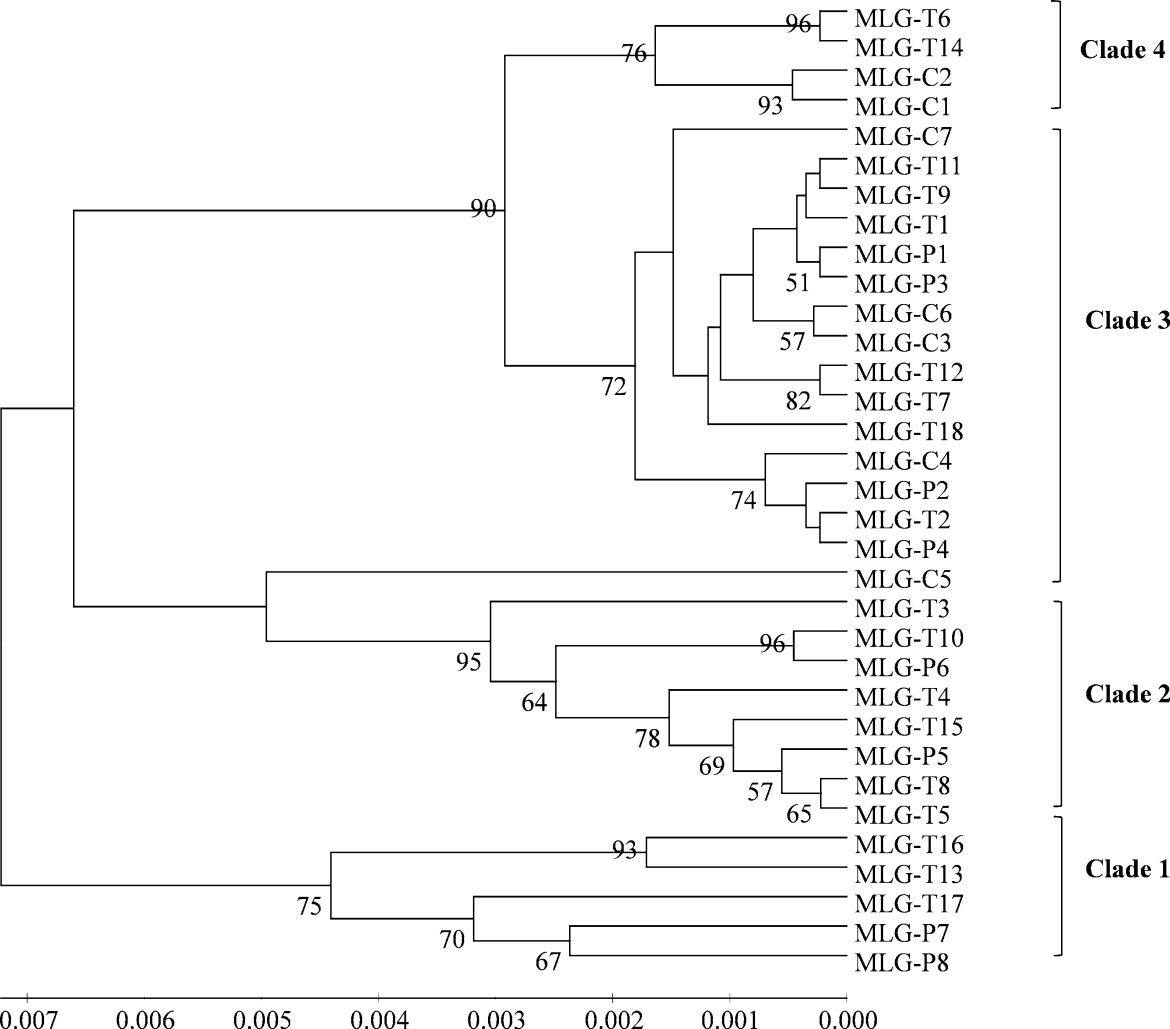
An unweighted pair-group mean average (UPGMA) tree for multilocus genotypes (MLGs) of ITS genotypes Type IV, Macaque3 and Peru8 of *Enterocytozoon bieneusi*. Bootstrap values greater than 50% from 1000 replicates are shown at the nodes

Maximum likelihood analysis of the concatenated sequences of the 33 MLGs showed four main subpopulations (SPs). SP1 contained samples of three MLGs of ITS genotype Type IV and two MLGs of ITS genotype Peru8, SP2 contained six MLGs of ITS genotype Type IV and two MLGs of ITS genotype Peru8, SP3 contained seven MLGs of ITS genotype Type IV, four MLGs of ITS genotype Peru8, and five MLGs of ITS genotype Macaque3, while SP4 contained two MLGs of ITS genotype Type IV and two MLGs of ITS genotype Macaque3 (Fig. 1). Similarly, four major clades were formed in UPGMA analysis of 33 MLGs in agreement with results of ML analysis of the concatenated sequence data (Fig. 2).

**Fig. 3:**
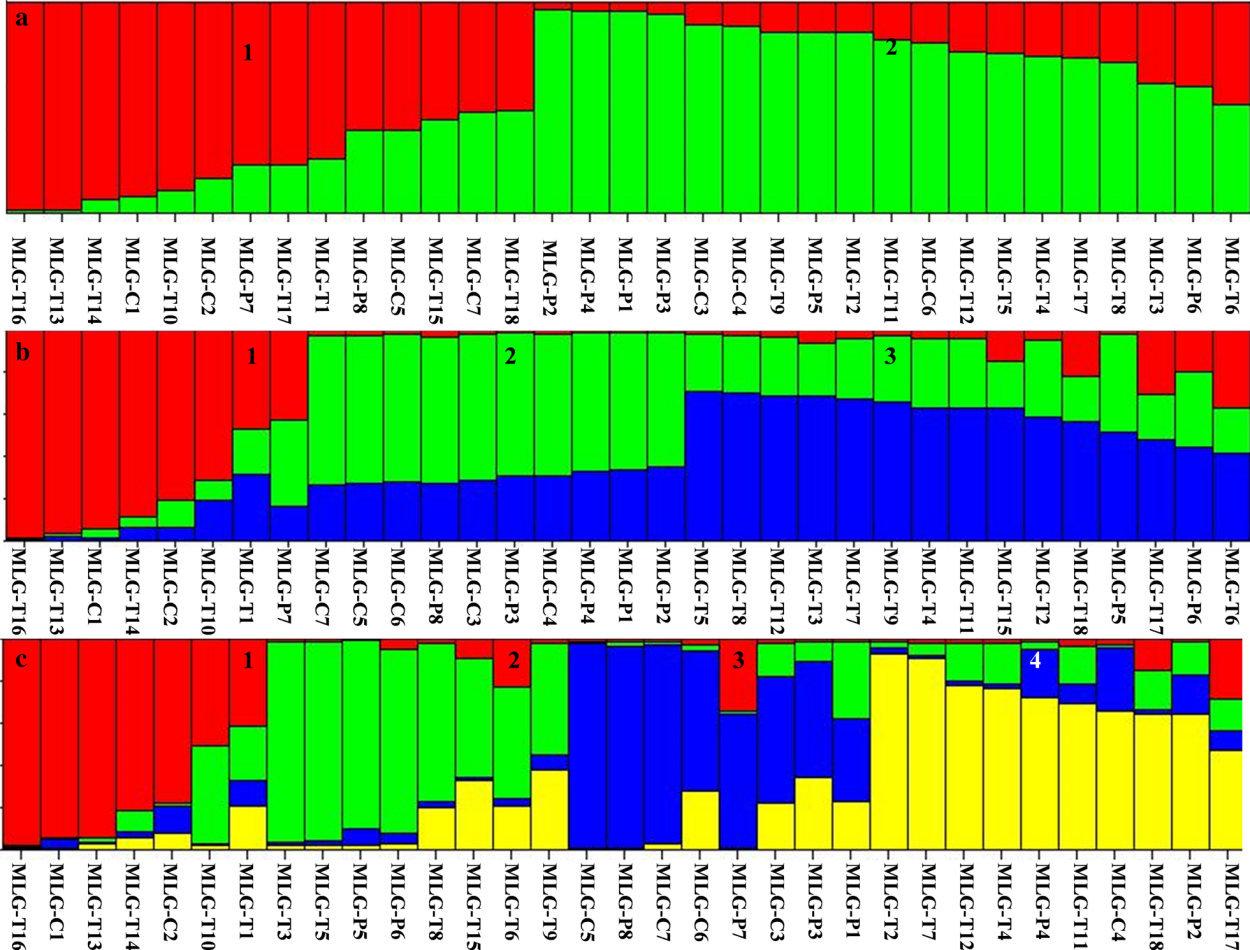
Subpopulation structure of 33 *Enterocytozoon bieneusi* MLGs. Different subpopulation patterns are shown depending on the setting of *K* values. **a** The generation of two subpopulations using *K* = 2. **b** The presence of three subpopulations using *K* = 3. **c** The appearance of four subpopulations using *K* = 4

In STRUCTURE analysis of the MLST data, we used the smallest value of *K* (*K* = 2) to measure population differentiation within the *E. bieneusi* population. Two subpopulations (SPs) with alpha (*α*) value of 0.104 were produced (1, 2; Fig. 3a). SP1 contained 14 MLGs, while SP2 contained the remaining 19 MLGs (Fig. 3a). When the *K* value was increased to 3, three clear SPs were formed (*α* = 0.074; Fig. 3b). SP1 included 8 MLGs belonging to SP1 at *K* = 2, SP2 included 3 MLGs belonging to SP1 and 7 MLGs belonging to SP2 at *K* = 2, while SP3 included 3 MLGs belonging to SP1 and 12 MLGs belonging to SP2 at *K* = 2 (Fig. 3a, b). When using *K* = 4, four clear SPs formed, with individuals in SP1 being similar to SP1 at *K* = 2 and 3 (Fig. 3). In addition, the *α* value of 0.061 was similar to the values generated at *K* = 2 and 3, suggesting the substructures at *K* = 4 could be realistic.

*F*_ST_ analysis was used to assess the sub-structuring within the *E. bieneusi* population. For SP1 and SP2 generated by STRUCTURE at *K* = 2, the differentiation was significant (*F*_ST_ = 0.161, *P* < 0.0001). At *K* = 3, the differentiation between SP1 and SP2 (*F*_ST_ = 0.362, *P* < 0.0001), SP1 and SP3 (*F*_ST_ = 0.274, *P* < 0.0001), and SP2 and SP3 (*F*_ST_ = 0.234, *P* < 0.0001) were also significant. Similarly at *K* = 4, the differentiation between SP1 and SP2 (*F*_ST_ = 0.349, *P* < 0.0001), SP1 and SP3 (*F*_ST_ = 0.345, *P* < 0.0001), SP1 and SP4 (*F*_ST_ = 0.313, *P* < 0.0001), SP2 and SP3 (*F*_ST_ = 0.293, *P* < 0.0001), SP2 and SP4 (*F*_ST_ = 0.233, *P* < 0.0001), and SP3 and SP4 (*F*_ST_ = 0.254, *P* < 0.0001) were all significant.

## Discussion

Currently, multilocus sequence typing has been used to analyze the genetic diversity of *E. bieneusi* around the world [15, 42, 43]. In Peru, India and Nigeria, 66 MLGs were identified in HIV-infected individuals [33, 37]. In China, 59 and 44 MLGs were identified in NHPs and pigs, respectively, while some genetic diversity based on MLST data was also observed in *E. bieneusi* isolates from domestic, wild, and zoo animals [29, 36, 42–48]. However, few studies analyzed the MLGs of this pathogen in NHPs in China. In the present study, 33 MLGs were identified from 53 *E. bieneusi*-positive samples from macaque monkeys, indicating a high genetic diversity within some common ITS genotypes (Type IV, Macaques3 and Peru8), and supporting high-resolution of the multilocus sequencing tool established by Feng et al. [32].

A strong and significant intragenic LD was observed in the analysis of the MLST data, indicating an overall clonal population structure of *E. bieneusi* in this study. In previous studies, the strong and significant intragenic LD obtained was used as an indication for the presence of a clonal population structure of *E. bieneusi* in the AIDS patients, giant pandas, pigs, fur animals and NHPs [36, 37, 47, 49, 50]. Therefore, there was a clonal endemicity of *E. bieneusi* in the study population.

Nevertheless, the MLGs found in crab-eating macaques were not segregated by ITS genotypes, indicating the existence of genetic recombination in the *E. bieneusi* population studied. In previous investigations, *E. bieneusi* MLGs were segregated by ITS genotypes for some genotypes with narrower host ranges, such as A, EbpA, SCO2 and Cs-4, but not for zoonotic genotypes such as Type IV, D, WL11 and Peru11 [15, 16]. Similarly, in this study, MLGs of *E. bieneusi* in crab-eating macaques were also not segregated by ITS genotypes, especially for the zoonotic genotypes Type IV and Peru8. Genetic recombination appears to be common in some microsporidia of insects, including *Kneallhazia solenopsae* in fire ants and *Andreanna caspii* in mosquitoes. In a previous study in China, the MLGs of some common ITS genotypes (Type IV, Peru8, D, Macaque3, CM2, Peru11, Henan V, PigEBITS7) in NHPs were not segregated by ITS genotypes in phylogenetic analysis, suggesting a possible occurrence of sexual recombination in *E. bieneusi* [36]. Therefore, data from both studies have indicated that there could be some sexual recombination among common *E. bieneusi* genotypes in NHPs.

Four SPs were obtained in this study among MLGs isolates within Group 1, according to results of phylogenetic and subpopulation genetics analyses. The four SPs all appear to have an epidemic population structure. In previous studies, the ITS genotype Macaque3 was mostly found in NHPs, and only occasionally in dogs [8–10, 26, 51]. Therefore, the clustering of the host-adapted ITS genotype Macaque3 in SP3 and SP4 suggests the NHP-specific nature of these two subpopulations. Both potentially zoonotic and host-adapted subpopulations have been identified in Group 1 in previous studies. The former included MLGs of ITS genotypes Type IV, D, Peru8, WL11, and Peru11 in humans, NHPs, pigs and companion animals, whereas the latter included MLGs of ITS genotypes A in humans, EbpA and CS-4 in pigs, and Macaque3 in NHPs [1, 15, 21, 36, 52, 53]. Therefore, SP3 and SP4 including MLGs of Macaque3 were potentially NHPs-adapted subpopulations, while SP1 and SP2 only including MLGs of ITS genotype Type IV and Peru8 were potentially zoonotic subpopulations.

## Conclusions

A much higher genetic diversity was revealed in *E. bieneusi* isolates from crab-eating macaques by multilocus sequence typing (33 MLGs) than by ITS genotyping (three ITS genotypes). This appears to have resulted from genetic recombination among common ITS genotypes despite an overall clonal population structure in *E. bieneusi*. Nevertheless, the MLGs generated from the analysis have formed four subpopulations with different host ranges and an epidemic population structure. These results provide data on the population and subpopulation structure of *E. bieneusi* in NHPs, and would improve our perception of *E. bieneusi* epidemiology in these animals.

## Data Availability

The data supporting the conclusions of this article are included within the article. Representative nucleotide sequences generated in this study were deposited in the GenBank database under the accession numbers MT137078-MT137095.

## Abbreviations

ITS: internal transcribed spacer
LD: linkage disequilibrium
LE: linkage equilibrium
ML: maximum likelihood
MLG: multi-locus genotype
NHP: non-human primate
PCR: polymerase chain reaction
Rms: recombination events
SP: subpopulation
UPGMA: un-weighted pair group mean average

## References

[1] Santin M, Fayer R (2011). Microsporidiosis: *Enterocytozoon bieneusi* in domesticated and wild animals. Res Vet Sci.

[2] Desportes I, Le Charpentier Y, Galian A, Bernard F, Cochand-Priollet B, Lavergne A (1985). Occurrence of a new microsporidan: *Enterocytozoon bieneusi* n.g., n. sp., in the enterocytes of a human patient with AIDS. J Protozool.

[3] Tavalla M, Mardani-Kateki M, Abdizadeh R, Nashibi R, Rafie A, Khademvatan S (2017). Molecular identification of *Enterocytozoon bieneusi* and *Encephalitozoon* spp. in immunodeficient patients in Ahvaz, Southwest of Iran. Acta Trop.

[4] Didier ES, Weiss LM (2011). Microsporidiosis: not just in AIDS patients. Curr Opin Infect Dis.

[5] Lobo ML, Xiao L, Antunes F, Matos O (2012). Microsporidia as emerging pathogens and the implication for public health: a 10-year study on HIV-positive and -negative patients. Int J Parasitol.

[6] Li W, Feng Y, Santin M (2019). Host specificity of *Enterocytozoon bieneusi* and public health implications. Trends Parasitol.

[7] Matos O, Lobo ML, Xiao L (2012). Epidemiology of *Enterocytozoon bieneusi* infection in humans. J Parasitol Res.

[8] Chen L, Zhao J, Li N, Guo Y, Feng Y, Feng Y (2019). Genotypes and public health potential of *Enterocytozoon bieneusi* and *Giardia duodenalis* in crab-eating macaques. Parasites Vectors.

[9] Karim MR, Dong H, Li T, Yu F, Li D, Zhang L (2015). Predomination and new genotypes of *Enterocytozoon bieneusi* in captive nonhuman primates in zoos in China: high genetic diversity and zoonotic significance. PLoS ONE.

[10] Yang H, Lin Y, Li Y, Song M, Lu Y, Li W (2017). Molecular characterization of *Enterocytozoon bieneusi* isolates in laboratory macaques in north China: zoonotic concerns. Parasitol Res.

[11] Zhong Z, Li W, Deng L, Song Y, Wu K, Tian Y (2017). Multilocus genotyping of *Enterocytozoon bieneusi* derived from nonhuman primates in southwest China. PLoS ONE.

[12] Mynarova A, Foitova I, Kvac M, Kvetonova D, Rost M, Morrogh-Bernard H (2016). Prevalence of *Cryptosporidium* spp., *Enterocytozoon bieneusi*, *Encephalitozoon* spp. and *Giardia intestinalis* in wild, semi-wild and captive orangutans (*Pongo abelii* and *Pongo pygmaeus*) on Sumatra and Borneo, Indonesia. PLoS ONE.

[13] Sak B, Petrzelkova KJ, Kvetonova D, Mynarova A, Pomajbikova K, Modry D (2014). Diversity of microsporidia, *Cryptosporidium* and *Giardia* in mountain gorillas (*Gorilla beringei beringei*) in Volcanoes National Park, Rwanda. PLoS ONE.

[14] Li W, Kiulia NM, Mwenda JM, Nyachieo A, Taylor MB, Zhang X (2011). *Cyclospora papionis*, *Cryptosporidium hominis*, and human-pathogenic *Enterocytozoon bieneusi* in captive baboons in Kenya. J Clin Microbiol.

[15] Li W, Xiao L (2019). Multilocus sequence typing and population genetic analysis of *Enterocytozoon bieneusi*: host specificity and its impacts on public health. Front Genet.

[16] Li W, Feng Y, Zhang L, Xiao L (2019). Potential impacts of host specificity on zoonotic or interspecies transmission of *Enterocytozoon bieneusi*. Infect Genet Evol.

[17] Ye J, Xiao L, Ma J, Guo M, Liu L, Feng Y (2012). Anthroponotic enteric parasites in monkeys in public park, China. Emerg Infect Dis.

[18] Zhang X, Wang Z, Su Y, Liang X, Sun X, Peng S (2011). Identification and genotyping of *Enterocytozoon bieneusi* in China. J Clin Microbiol.

[19] Du S, Zhao G, Shao J, Fang Y, Tian G, Zhang L (2015). *Cryptosporidium* spp., *Giardia intestinalis*, and *Enterocytozoon bieneusi* in captive non-human primates in Qinling mountains. Korean J Parasitol.

[20] Sak B, Petrzelkova KJ, Kvetonova D, Mynarova A, Shutt KA, Pomajbikova K (2013). Long-term monitoring of microsporidia, *Cryptosporidium* and *Giardia* infections in western lowland gorillas (*Gorilla gorilla gorilla*) at different stages of habituation in Dzanga Sangha Protected Areas, Central African Republic. PLoS ONE.

[21] Karim MR, Wang R, Dong H, Zhang L, Li J, Zhang S (2014). Genetic polymorphism and zoonotic potential of *Enterocytozoon bieneusi* from nonhuman primates in China. Appl Environ Microbiol.

[22] Wang S, Wang R, Fan X, Liu T, Zhang L, Zhao G (2018). Prevalence and genotypes of *Enterocytozoon bieneusi* in China. Acta Trop.

[23] Lores B, Lopez-Miragaya I, Arias C, Fenoy S, Torres J, del Aguila C (2002). Intestinal microsporidiosis due to *Enterocytozoon bieneusi* in elderly human immunodeficiency virus-negative patients from Vigo, Spain. Clin Infect Dis.

[24] Wang L, Zhang H, Zhao X, Zhang L, Zhang G, Guo M (2013). Zoonotic *Cryptosporidium* species and *Enterocytozoon bieneusi* genotypes in HIV-positive patients on antiretroviral therapy. J Clin Microbiol.

[25] Santin M, Fayer R (2009). *Enterocytozoon bieneusi* genotype nomenclature based on the internal transcribed spacer sequence: a consensus. J Eukaryot Microbiol.

[26] Karim MR, Dong H, Yu F, Jian F, Zhang L, Wang R (2014). Genetic diversity in *Enterocytozoon bieneusi* isolates from dogs and cats in China: host specificity and public health implications. J Clin Microbiol.

[27] Luo R, Xiang L, Liu H, Zhong Z, Liu L, Deng L (2019). First report and multilocus genotyping of *Enterocytozoon bieneusi* from Tibetan pigs in southwestern China. Parasite.

[28] Zou Y, Hou J, Li F, Zou F, Lin R, Ma J (2018). Prevalence and genotypes of *Enterocytozoon bieneusi* in pigs in southern China. Infect Genet Evol.

[29] Tang C, Cai M, Wang L, Guo Y, Li N, Feng Y (2018). Genetic diversity within dominant *Enterocytozoon bieneusi* genotypes in pre-weaned calves. Parasites Vectors.

[30] Tian G, Zhao G, Du S, Hu X, Wang H, Zhang L (2015). First report of *Enterocytozoon bieneusi* from giant pandas (*Ailuropoda melanoleuca*) and red pandas (*Ailurus fulgens*) in China. Infect Genet Evol.

[31] Deng L, Li W, Yu X, Gong C, Liu X, Zhong Z (2016). First report of the human-pathogenic *Enterocytozoon bieneusi* from red-bellied tree squirrels (*Callosciurus erythraeus*) in Sichuan, China. PLoS ONE.

[32] Feng Y, Li N, Dearen T, Lobo ML, Matos O, Cama V (2011). Development of a multilocus sequence typing tool for high-resolution genotyping of *Enterocytozoon bieneusi*. Appl Environ Microbiol.

[33] Li W, Cama V, Feng Y, Gilman RH, Bern C, Zhang X (2012). Population genetic analysis of *Enterocytozoon bieneusi* in humans. Int J Parasitol.

[34] Desoubeaux G, Nourrisson C, Moniot M, De Kyvon MA, Bonnin V, De La Bretonniere ME (2019). Genotyping approach for potential common source of *Enterocytozoon bieneusi* infection in hematology unit. Emerg Infect Dis.

[35] Widmer G, Dilo J, Tumwine JK, Tzipori S, Akiyoshi DE (2013). Frequent occurrence of mixed *Enterocytozoon bieneusi* infections in humans. Appl Environ Microbiol.

[36] Karim MR, Wang R, He X, Zhang L, Li J, Rume FI (2014). Multilocus sequence typing of *Enterocytozoon bieneusi* in nonhuman primates in China. Vet Parasitol.

[37] Li W, Cama V, Akinbo FO, Ganguly S, Kiulia NM, Zhang X (2013). Multilocus sequence typing of *Enterocytozoon bieneusi*: lack of geographic segregation and existence of genetically isolated sub-populations. Infect Genet Evol.

[38] Rozas J, Sanchez-DelBarrio JC, Messeguer X, Rozas R (2003). DnaSP, DNA polymorphism analyses by the coalescent and other methods. Bioinformatics.

[39] Excoffier L, Lischer HE (2010). Arlequin suite ver 3.5: a new series of programs to perform population genetics analyses under Linux and Windows. Mol Ecol Resour.

[40] Haubold B, Hudson RR (2000). LIAN 3.0: detecting linkage disequilibrium in multilocus data. Linkage Analysis. Bioinformatics.

[41] Falush D, Stephens M, Pritchard JK (2003). Inference of population structure using multilocus genotype data: linked loci and correlated allele frequencies. Genetics.

[42] Gui B, Zou Y, Chen Y, Li F, Jin Y, Liu M (2020). Novel genotypes and multilocus genotypes of *Enterocytozoon bieneusi* in two wild rat species in China: potential for zoonotic transmission. Parasitol Res.

[43] Zhang N, Wu R, Ji T, Cui L, Cao H, Li D (2020). Molecular detection, multilocus genotyping, and population genetics of *Enterocytozoon bieneusi* in pigs in southeastern China. J Eukaryot Microbiol.

[44] Ma Y, Zou Y, Liu Q, Xie S, Li R, Zhu X (2019). Prevalence and multilocus genotypes of *Enterocytozoon bieneusi* in alpacas (*Vicugna pacos*) in Shanxi Province, northern China. Parasitol Res.

[45] Zhao G, Du S, Wang H, Hu X, Deng M, Yu S (2015). First report of zoonotic *Cryptosporidium* spp., *Giardia intestinalis* and *Enterocytozoon bieneusi* in golden takins (*Budorcas taxicolor bedfordi*). Infect Genet Evol.

[46] Deng L, Li W, Zhong Z, Gong C, Liu X, Huang X (2016). Molecular characterization and multilocus genotypes of *Enterocytozoon bieneusi* among horses in southwestern China. Parasites Vectors.

[47] Li W, Wan Q, Yu Q, Yang Y, Tao W, Jiang Y (2016). Genetic variation of mini- and microsatellites and a clonal structure in *Enterocytozoon bieneusi* population in foxes and raccoon dogs and population differentiation of the parasite between fur animals and humans. Parasitol Res.

[48] Wang X, Wang R, Ren G, Yu Z, Zhang L, Zhang S (2016). Multilocus genotyping of *Giardia duodenalis* and *Enterocytozoon bieneusi* in dairy and native beef (Qinchuan) calves in Shaanxi Province, northwestern China. Parasitol Res.

[49] Li W, Song Y, Zhong Z, Huang X, Wang C, Li C (2017). Population genetics of *Enterocytozoon bieneusi* in captive giant pandas of China. Parasites Vectors.

[50] Wan Q, Xiao L, Zhang X, Li Y, Lu Y, Song M (2016). Clonal Evolution of *Enterocytozoon bieneusi* populations in swine and genetic differentiation in subpopulations between isolates from swine and humans. PLoS Negl Trop Dis.

[51] Zhao W, Zhou H, Jin H, Sun L, Li P, Liu M (2020). Genotyping of *Enterocytozoon bieneusi* among captive long-tailed macaques (*Macaca fascicularis*) in Hainan Province: high genetic diversity and zoonotic potential. Acta Trop.

[52] Thellier M, Breton J (2008). *Enterocytozoon bieneusi* in human and animals, focus on laboratory identification and molecular epidemiology. Parasite.

[53] Li D, Zheng S, Zhou C, Karim MR, Wang L, Wang H (2019). Multilocus typing of *Enterocytozoon bieneusi* in pig reveals the high prevalence, zoonotic potential, host adaptation and geographical segregation in China. J Eukaryot Microbiol.

